# The kynurenine pathway in traumatic brain injuries and concussion

**DOI:** 10.3389/fneur.2023.1210453

**Published:** 2023-06-09

**Authors:** Mona Dehhaghi, Benjamin Heng, Gilles J. Guillemin

**Affiliations:** Neuroinflammation Group, Department of Biomedical Sciences, Macquarie University, Sydney, NSW, Australia

**Keywords:** the kynurenine pathway, neuroinflammation, traumatic brain injury, quinolinic acid, inflammation

## Abstract

Up to 10 million people per annum experience traumatic brain injury (TBI), 80–90% of which are categorized as mild. A hit to the brain can cause TBI, which can lead to secondary brain injuries within minutes to weeks after the initial injury through unknown mechanisms. However, it is assumed that neurochemical changes due to inflammation, excitotoxicity, reactive oxygen species, etc., that are triggered by TBI are associated with the emergence of secondary brain injuries. The kynurenine pathway (KP) is an important pathway that gets significantly overactivated during inflammation. Some KP metabolites such as QUIN have neurotoxic effects suggesting a possible mechanism through which TBI can cause secondary brain injury. That said, this review scrutinizes the potential association between KP and TBI. A more detailed understanding of the changes in KP metabolites during TBI is essential to prevent the onset or at least attenuate the severity of secondary brain injuries. Moreover, this information is crucial for the development of biomarker/s to probe the severity of TBI and predict the risk of secondary brain injuries. Overall, this review tries to fill the knowledge gap about the role of the KP in TBI and highlights the areas that need to be studied.

## 1. Introduction

Traumatic brain injury (TBI) is a major cause of disability and death worldwide which affects about 10 million people annually across all age groups (1). According to the Centers for Disease Control and Prevention (CDC), in 2019, more than 223,000 TBI-associated cases were hospitalized in the USA (2). Generally, TBI can be classified as mild, moderate, and severe according to the Glasgow Coma Scale (GCS) score and neurobehavioral characteristics after injury. The most TBIs reported are categorized as mild TBI or concussions (80–90% of cases) that are caused by a bump or blow to the head as well as by a strong hit to the body that leads to quick back and forth movements in the head and brain followed by bouncing the brain, chemical alteration in the brain, and damaging brain cells (3–5). TBI causes a wide range of symptoms including headache, dizziness, fatigue, vomiting, cognitive impairment, loss of concentration, memory deficits, loss of consciousness, and mood swings. TBI can also be classified based on the injury progression to primary and secondary injuries. Primary injury can be caused by immediate physical injury to the brain and may follow by contusion, concussion, and disruption of brain tissue. The secondary injury occurs due to the primary injury's biomolecular changes and pathophysiological consequences. The secondary injury may emerge from minutes to weeks after the initial injury and is known by cerebral oedema, hemorrhage, diffuse brain swelling, infection, and ischemia (6–8). The pathophysiology of TBI has been investigated in several studies (9–11). Briefly, after primary brain injury, some neurochemical changes occur that lead to secondary injuries. Among them, releasing excitatory amino acids (*e.g.*, glutamate) and generating reactive oxygen species (ROS) and nitric oxide are closely associated with brain cell injury. Increased calcium influx to the brain cells induced by excitatory amino acids could promote ROS generation in neurons. Excessive amounts of intracellular calcium and ROS induce nitric oxide release, leading to oxidative stress, lipid peroxidation, and the release of excitatory amino acids. Glutamate exerts its excitatory function in TBI through alterations in presynaptic and postsynaptic glutamate receptors (12, 13). Generally, glutamate receptors (i.e., amino-3-hydroxy-5-methyl-4-isoxazole propionic acid (AMPA) receptors and N-methyl-D-aspartic acid (NMDA) receptors) promote the influx of calcium into the neuronal cells in TBI. It has been shown that employing NMDA receptor antagonists could improve TBI symptoms (14–16). One of the main pathophysiological processes which play critical roles in clinical and functional outcomes in TBI is inflammation. TBI promotes activation of resident microglia and peripheral neutrophil recruitment subsequently followed by infiltration of macrophages and lymphocytes as well as the release of inflammatory cytokines and chemokines (17, 18). One of the most important pathways activated during inflammation is the kynurenine pathway (KP). Under the inflammatory condition, KP metabolites could be involved in excitotoxicity through the production of neurotoxic compounds such as quinolinic acid (QUIN), kynurenine (KYN), and 3-hydroxykynurenine (3HK) (19–21). KP metabolites play different roles including neurotoxic, neuroprotective, and immunomodulatory activities contributing to various neuroinflammatory-associated diseases such as Alzheimer's disease (22, 23), amyotrophic lateral sclerosis (24), and multiple sclerosis (25, 26). In this review, we proposed that dysregulation of the KP could be a potential underlying mechanism of TBI-induced neurotoxicity.

Moreover, this review addresses the evidence supporting the involvement of the KP in TBI pathophysiology. This review discusses the role of KP metabolites in the development of secondary brain injuries. The first section describes the KP functions and regulations. This is followed by hypothesizing possible mechanisms of KP involvement in TBI supported by the body of evidence extracted from the literature. Understanding the possible association between the KP and TBI can be helpful in exploring biomarkers as well as new treatment targets.

## 2. The kynurenine pathway

**Figure 1:**
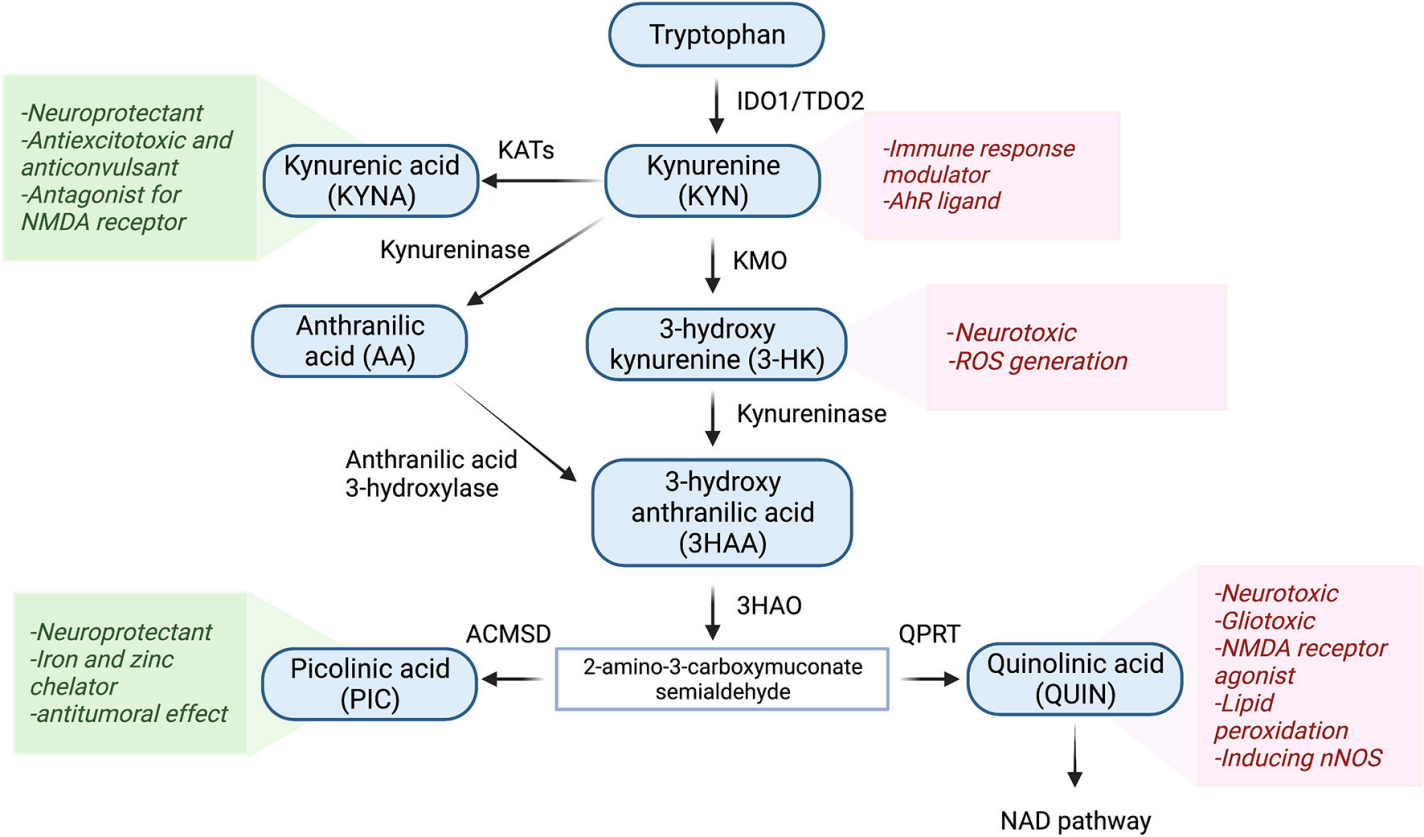
Schematic representation of the kynurenine pathway. Tryptophan (>90%) is mainly metabolized through the KP. In the first step of the KP, tryptophan is cleaved either by indoleamine 2,3-dioxygenase 1 (IDO-1), IDO-2, or tryptophan 2,3-dioxygenase (TDO), producing various bioactive intermediates such as excitotoxins quinolinic acid (QUIN), 3-hydroxykynurenine, and the neuroprotective metabolites kynurenic acid (KYNA) and picolinic acid (PIC). Ultimately, the KP leads to the de novo synthesis pathway of NAD^+^ which is linked to cellular energy. IDO1, indoleamine 2 3-dioxygenase; TDO2, tryptophan 2 3-dioxygenase; KMO, kynurenine 3-monooxygenase; KAT, kynurenine aminotransferase; 3HAO, 3-hydroxy anthranilic acid oxygenase; ACMSD, aminocarboxymuconate semialdehyde decarboxylase; QPRT, Quinolinic acid phosphoribosyl transferase.

The KP is the main route of tryptophan metabolism synthesizing nicotinamide adenosine dinucleotide (NAD^+^) as well as several neuroactive metabolites (27–29) (Figure 1). About 95% of tryptophan is metabolized to KYN through the KP by the activity of regulatory enzymes indoleamine 2,3-dioxygenase-1 (IDO1) and tryptophan dioxygenase (TDO2). While IDO1 is predominantly expressed in macrophages, microglia, astrocytes, and neuronal cells, TDO2 is primarily expressed in the liver (30, 31). Several inflammatory cytokines such as interferon-gamma (INF-γ), interleukin 1β (IL-1β), and tumor necrosis factor-α (TNF-α) induce the activity of IDO1 (21, 32). Conversion of tryptophan to KYN follows by production of neuroprotective and neurotoxic metabolites. KYN is converted to kynurenine acid (KYNA) by kynurenine aminotransferases (KATs) or 3-HK through kynurenine-3-monooxygenase (KMO). The latter eventuates to the generation of the neurotoxic metabolite, QUIN. The KP has been identified in most cells, however, it is highly activated in monocytic cells under inflammatory circumstances (33). During the inflammation, activated macrophages and microglia produce QUIN which can further decrease KYNA by eliminating astrocytes. It has been demonstrated that 500–1,200 nM of QUIN induces apoptosis in human astrocytes (34, 35).

Among KP metabolites, KYNA and QUIN due to their polar properties and lack of transportation systems cannot cross the blood-brain barrier (BBB), while other KP metabolites cross the BBB through active transportation and passive diffusion (36, 37). Among the neuroactive KP metabolites, QUIN is assumed to be an important molecule in TBI. It has been documented that the level of QUIN is strongly elevated during inflammation (32, 38, 39). Moreover, QUIN can act as a glutamate agonist and activate the NMDA receptor which stimulates calcium influx into the neurons and ultimately promotes cell deaths (40, 41).

### 2.1. Involvement of the KP in TBI

**Figure 2:**
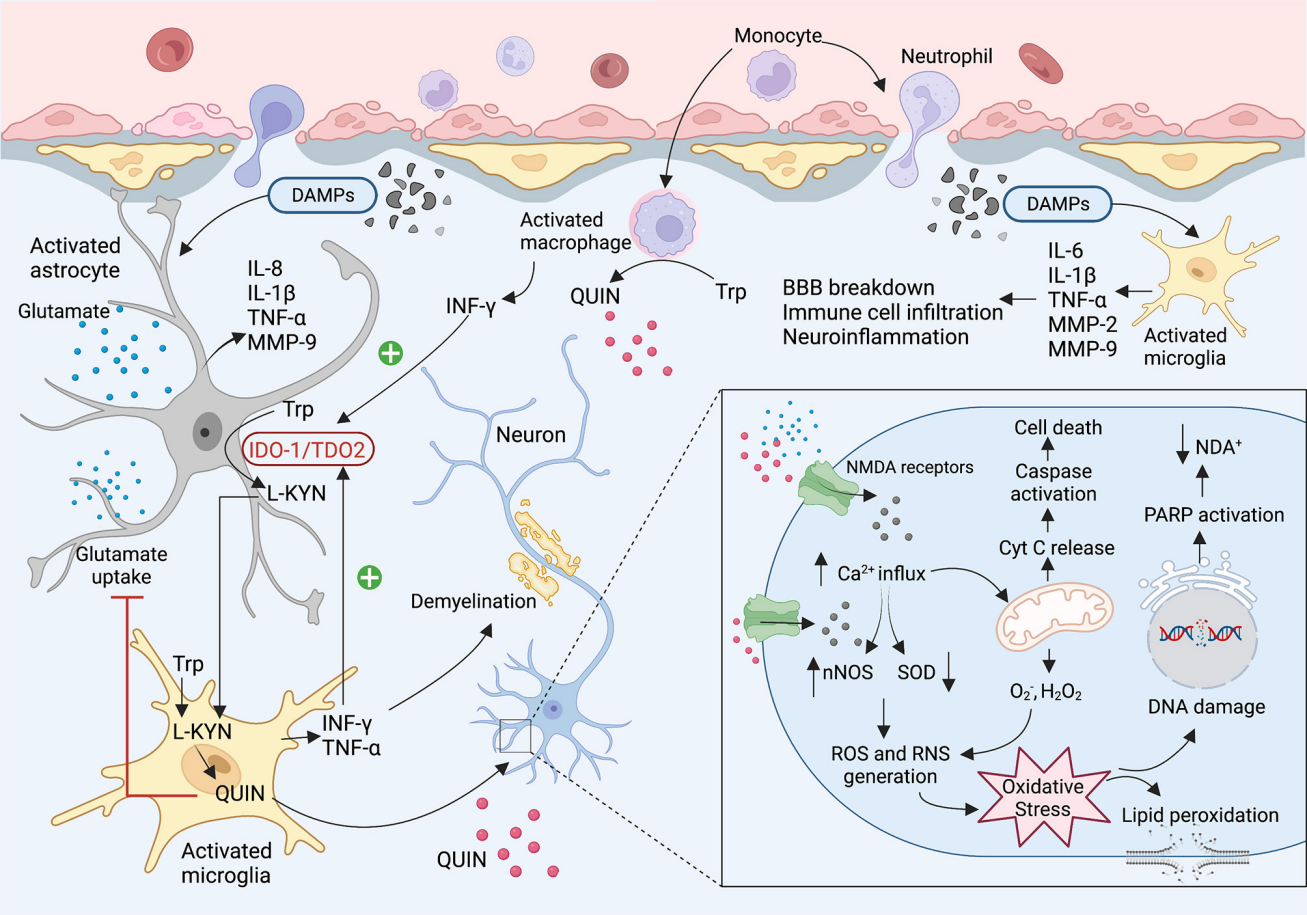
The KP involvement in the TBI and concussion. After brain injury, damaged tissues start to release DMAPs as an early stimulator of the resident cells. The DMAPs are recognized by activated microglia and astrocytes that lead to releasing several cytokines and chemokines. Elevated levels of INF-γ and TNF-α activate the IDO1 leading to the production of KP neuroactive metabolites. Activated microglia and infiltrating macrophages are known as the main cells to produce QUIN in the brain. QUIN exerts its excitotoxicity through various mechanisms. QUIN is a glutamate agonist which can activate the NMDA receptor. Upon activation of NMDAR, a significant increase in intracellular Ca^2+^ influx occurs that leads to ROS and RNS generation, lipid peroxidation, mitochondrial dysfunction, DNA damage, and ultimately cell death.

In the early phase of brain injury, damage-associated molecular patterns (DAMPs) are released as primary inducers of the innate immune system. These molecules are recognized by Toll-Like Receptors (TLR) expressing cells such as macrophages, astrocytes, dendritic cells, and glial cells which results in infiltrating immune cells to the brain, triggering an inflammatory response, and releasing cytokines and chemokines (42–44). It is well known that IDO1/TDO2 (i.e., the first enzyme of the KP) is highly activated during the inflammation leading to an increase in levels of neurotoxic metabolites such as 3-HK and QUIN. In the central nervous system (CNS), most cells possess all the enzymes of the KP and can catabolise tryptophan, however, human neurons, astrocytes, and oligodendrocytes cannot produce QUIN. This neurotoxic metabolite is mainly synthesized by activated microglia and infiltrating macrophages (27). QUIN as an NMDA receptor antagonist can activate the receptor contributing to increased Ca^2+^ influx, inducing neuronal nitric oxide synthase (nNOS), and oxidative stress. Moreover, QUIN can increase glutamate release from the neuron and inhibits glutamate uptake by astrocytes resulting in glutamate accumulation, neurotoxicity, and cell death (41, 45, 46) (Figure 2).

Over the past decades, it has been established that inflammation, oxidative stress, and excitotoxicity play critical roles in TBI, however, the underlying mechanisms of secondary brain injury remain uncovered. Dysregulated KP and neuroinflammation have been identified in several CNS-associated diseases (e.g., Alzheimer's disease, amyotrophic lateral sclerosis, AIDS-dementia complex, and Parkinson's disease) (23, 24, 47, 48). Activation of the KP during the inflammation has raised the possibility of KP metabolites' involvement, particularly neurotoxic molecules such as QUIN, in secondary brain injury. One of the first studies investigating the KP dysregulation following TBI in humans was conducted by Sinz et al. (49). They examined cerebrospinal fluid (CSF) in 39 patients (GCS<8) during the first week of the injury. The levels of QUIN in patients significantly increased to 463±128 nmol/L (5–50-fold), about nine times higher than the normal concentration of QUIN in CSF, 72–83 h after injury. Their results did not show any relevance between QUIN concentration in patients and GCS, age, gender, and treatment. Elevated levels of QUIN in human CSF were reported in further studies. For instance, Yan et al. (19) measured KP metabolites in CSF from 28 TBI patients with severe TBI (GCS ≤ 8) from admission to 5 days after primary injury. Post-mortem brains were also collected to quantify the KP enzymes. Their results indicated an increase in the levels of KYN, KYNA and QUIN in CSF, while no significant changes were found in concentrations of tryptophan, AA, and 3-hydroxyanthranilic acid (3HAA). It is important to note that QUIN levels in patients' CSF were significantly higher than KYNA and KYN, suggesting elevated neurotoxicity in TBI cases.

Moreover, there was an inverse correlation between the levels of QUIN in CSF and the Glasgow Outcome Scale Extended (GOSE) score. Upregulating the IDO1 and kynureninase (KYNase) was also identified in the injured brains, supporting an increase in the levels of QUIN in CSF. It has been previously reported that the levels of pro-inflammatory molecules, particularly IL-6, IFN-γ, TNF-α, and GM-CSF, are significantly increased in TBI within early minutes of injury, suggesting an immediate inflammatory response (50). Both IFN-γ and TNF-α induce IDO1 and tryptophan degradation through the KP, contributing release of QUIN. The neurotoxic activity of QUIN occurs through its interaction with NMDA receptors contributing to Ca^2+^ influx and oxidative stress that ultimately leads to ecotoxicity and cell death (41).

In a recent study, Zhang et al. (51) studied the expression of the two KP enzymes, kynureninase and kynurenine-3-monooxygenase (KMO) and kynureninase, in animal models. Blast-related traumatic brain injury was induced in rats using a shock tube and the expression levels of KMO and kynureninase were assessed using immunohistochemistry. They found that the expression of kynureninase and KMO significantly increased within a week in the hippocampus of the animals with TBI compared to the control. *In vitro* and *in vivo* studies showed that using Ro 61-8048 as a KMO inhibitor significantly decreased the apoptotic rate of neurons in the hippocampal CA1 area and improved the behavior of rats. However, benserazide hydrochloride (i.e., kynureninase inhibitor) treatment only showed protective effects *in vitro* (51). Decreased KYNA/QUIN and increased levels of QUIN in cases with sport-related concussion (SRC) have been reported previously (52).

Moreover, it has been reported that the concentration of QUIN in the plasma of football players with a prior concussion was higher than the control group even 10 months post-injury (53). It is inconsistent with the further study of Meier et al. (54) which reported that high school football players with a history of concussion had high levels of QUIN in serum up to 45 days after the injury in comparison to those who had no prior concussion, suggesting that a history of concussion may affect the KP metabolism toward QUIN production with a constant elevated level even after days or months. In a recent study, Meier et al. (55) reported an association between KP metabolites and functional connectivity in football players with a history of concussion. Accordingly, a positive correlation was found between QUIN and connectivity strength in football players with acute injury and a history of concussion. At the same time this association was not observed in cases suffering acute injury without a prior concussion or healthy control group. Hypothetically, elevated levels of QUIN in individuals with concussion promote glutamatergic dysfunction that finally induces altered functional connectivity and psychiatric disorders (55). Table 1 summarizes the studies investigating the alterations of kynurenine pathway metabolites in TBI and SRC cases.

**Table 1:** A summary of measurement of kynurenine pathway metabolites and enzymes in cases with TBI and SRC.

**Type of injury**	**Sample origin**	**Patients/animal model**	**Metabolite/enzyme**	**Time of assessment**	**Results**	**References**
TBI	CSF	39 patients (GCS < 8)	QUIN	72–83 h post-injury	The levels of QUIN increased to 463 ± 128 nmol/L (5–50-fold) compared to control	(49)
TBI	CSF, post-mortem brains	28 patients (GCS ≤ 8)	KYN, KYNA, QUIN, Trp, AA, 3-HAA	From admission to 5 days post-injury	- Increased levels of KYN, QUIN, KYNA - No significant changes in levels of Trp, AA, and 3-HAA - Upregulating IDO-1 and KYNase in brain tissue - Increased levels of IL-6, IFN-γ, TNF-α, and GM-CSF	(19)
SRC	Plasma	16 concussed cases	KYN, KYNA, QUIN	1 day, 1 week, 1-month post-injury	- Decreased KYNA/QUIN - increased levels of QUIN	(52)
SRC	Serum	59; High school football players	Trp, KYN, KYNA, QUIN, 3-HK	6 h, 24–48 h, 8–45 days post-injury	- Lower KYNA/QUIN in players with prior concussion - Increased KYNA/3-HK following concussion - Elevated QUIN levels in cases both acute and prior concussion	(54)
SRC	Serum	16 concussed cases without prior concussion, 21 concussed cases with prior concussion	Trp, KYN, KYNA, QUIN, 3-HK	1 day, 8 days 15 days, and 45 days post-injury	- QUIN levels were correlated with resting state fMRI in concussed participants with prior concussion	(55)
SRC	Hippocampal CA1 tissue	Rat	KMO and kynureninase	One week post injury	- Increased expression of kynureninase and KMO in the hippocampus of the animals - Ro 61-8048 as a KMO inhibitor decreased the apoptotic rate of neurons	(51)

### 2.2. Involvement of the KP in TBI-associated psychiatric disorders

TBI could be accompanied with various symptoms that are associated with cognitive impairment (memory and concentration problems) and psychiatric disorders (depression, anxiety, mood disorders) (56–58). Abnormally elevated levels of glutamate following TBI contribute to neuronal death and has been also linked to psychiatric conditions, cognitive impairment, and mood disorders (57–60). Since QUIN is known as an agonist for NMDA receptor, its increased levels under inflammatory conditions followed by severe TBI could over stimulate the NMDA receptor and induce neurotoxicity. Previous studies have reported that high levels of QUIN in blood and CSF could be associated with cytokine-induced depression and major depressive disorder (MDD) (61–63). On the other hand, increased levels of QUIN could be accompanied with either increasing or decreasing the levels of KYNA. Although KYNA is known as a neuroprotective metabolite and acts as antagonist for NMDA receptor, can induce glutamatergic hypofunction at higher levels leading to cognition impairment (64–66). In addition to the glutamatergic system, the cholinergic system has been involved in pathophysiology of TBI as well as further psychiatric complications. KYNA is also a non-competitive antagonist for α7 nicotinic acetylcholine receptors (α7nAChRs), ion channels widely expressed in central nervous system that exert anti-inflammatory effects (67–69). Since cholinergic transmission mediated by α7nAChRs has critical roles in cognitive functions and anti-depressant effects, inhibition of α7nAChRs by KYNA could be associated with development of psychiatric disorders.

## 3. Clinical implications

Considering dysregulation of the KP in inflammatory diseases, KP enzymes can be considered as plausible therapeutic targets for alleviating the neuroinflammation, neuropathological, and psychiatric symptoms of TBI and concussion. Therapeutic approaches have designed to target the KP enzymes, particularly IDO-1, to prevent inflammation and depressogenic effects (70–72). Theoretically, inhibition of KMO or increasing the activity of KATs could be a potential approach to reduce the synthesis of neurotoxic metabolites and decrease the neuroinflammation after brain injury. Due to QUIN activates the NMDA receptor, the next approach was to examine whether blocking the NMDA receptor alleviates inflammation-mediated depression. Ketamine, an antagonist of the NMDA receptor, was considered for this purpose. It has gained attentions as one of the most important therapeutic drugs for treatment-resistant depression (73, 74). Both clinical and pre-clinical evidence have documented that ketamine increased KYNA and decreased QUIN and 3-HK, suggesting activation of KAT compared to KMO and shifting the KP toward synthesis of neuroprotective metabolites rather than neurotoxic compounds (74). KMO has been previously reported critical for inflammation-mediated depressogenic effects in rodents (75). KYNA analog 4-Chlorokynurenine (AV-101) is another antagonist of the NMDA receptor. 4-Chlorokynurenine could easily cross the BBB and convert to 7-Chlorokynurenic acid in the brain by astrocytes (76). A single treatment with 4-Chlrokynurenine showed a relatively similar dose-dependent and persistent anti-depressant activity to ketamine in male mice (76). However, clinical trials failed to prove any improvement in depression in human (ClinicalTrials.gov Identifier: NCT02484456; ClinicalTrials.gov Identifier: NCT03078322).

## 4. Conclusions

TBI patients suffer from high mortality and morbidity due to a lack of effective treatment. Despite substantial developments in understanding the pathophysiology of TBI, the underlying mechanisms of this disease remain unclear. Dysregulation of the KP during a wide range of inflammatory diseases including TBI has received remarkable attention. However, there are still limited evidence investigating KP metabolites in acute TBI and second brain injury. Although alterations in all KP metabolites may not be specific to TBI, some neurotoxic molecules such as QUIN might be a potential biomarker to monitor the second injury and its outcome during a time. According to the related studies, it could be presumed that TBI may selectively induce the KP toward the production of QUIN, a prominent excitotoxic metabolite of the pathway. A possible association between TBI and levels of QUIN have raised the possibility of using treatments to suppress the KP enzymes to inhibit the neurotoxic effect of KP metabolites in secondary brain injury. One of the potential targets is KMO which converts KYN to 3-HK. Inhibiting KMO activity could decrease the levels of neurotoxic metabolites and increase KYNA production as a neuroprotective compound.

## Author contributions

MD and GG contributed to conception of the manuscript. MD wrote the manuscript and prepared the figures. GG and BH read and edited the manuscript. All authors approved the submitted version.

## References

[1] Maas AI Menon DK Adelson PD Andelic N Bell MJ Belli A. Traumatic brain injury: integrated approaches to improve prevention, clinical care, and research. Lancet Neurol. (2017) 16:987–1048. 10.1016/S1474-4422(17)30371-X 29122524

[2] CDC. “Traumatic Brain Injury & Concussion,” in Centers for Disease Control and Prevention, National Center for Injury Prevention and Control. (2022). Available online at: https://www.cdc.gov/traumaticbraininjury/index.html

[3] CDC. Report to Congress on Mild Traumatic Brain Injury in the United States: Steps to Prevent a Serious Public Health Problem. Atlanta, GA: Centers for Disease Control and Prevention (2003)

[4] Blennow K Brody DL Kochanek PM Levin H Mckee A Ribbers GM. Traumatic brain injuries. Nat Rev Dis Primers. (2016) 2:1–19. 10.1038/nrdp.2016.84 27853132

[5] Lumba-Brown A Yeates KO Sarmiento K Breiding MJ Haegerich TM Gioia GA. Centers for Disease Control and Prevention guideline on the diagnosis and management of mild traumatic brain injury among children. JAMA Pediatr. (2018) 172:e182853–e182853. 10.1001/jamapediatrics.2018.2853 30193284 PMC7006878

[6] Maas AI Stocchetti N Bullock R. Moderate and severe traumatic brain injury in adults. Lancet Neurol. (2008) 7:728–41. 10.1016/S1474-4422(08)70164-9 18635021

[7] Mustafa AG Alshboul OA. Pathophysiology of traumatic brain injury. Neurosci J. (2013) 18:222−35. 23887212

[8] Begasse De Dhaem O Robbins MS. Cognitive impairment in primary and secondary headache disorders. Curr Pain Headache Rep. (2022) 26:391–404. 10.1007/s11916-022-01039-5 35239156 PMC8891733

[9] Werner C Engelhard K. Pathophysiology of traumatic brain injury. Br J Anaesthesia. (2007) 99:4–9. 10.1093/bja/aem131 17573392

[10] Chakraborty R Tabassum H Parvez S. NLRP3 inflammasome in traumatic brain injury: Its implication in the disease pathophysiology and potential as a therapeutic target. Life Sci. (2022) 121352. 10.1016/j.lfs.2022.121352 36592789

[11] Mcdonald B. Effect of Injury Mechanism and Severity on the Molecular Pathophysiology of Traumatic Brain Injury. University of Nebraska, Biological Systems Engineering-Dissertations, Theses, and Student Research (2022).

[12] Greve MW Zink BJ. Pathophysiology of traumatic brain injury. Mt Sinai J Med. (2009) 76:97–104. 10.1002/msj.20104 19306379

[13] Giza CC Hovda DA. The pathophysiology of traumatic brain injury. In: Traumatic Brain Injury in Sports. Oxfordshire**:** Taylor & Francis. (2020) p. 45–70. 10.1201/9780367810535-4

[14] Kraus MF Smith G Butters M Donnell A Dixon E Yilong C. Effects of the dopaminergic agent and NMDA receptor antagonist amantadine on cognitive function, cerebral glucose metabolism and D2 receptor availability in chronic traumatic brain injury: a study using positron emission tomography (PET). Brain Injury. (2005) 19:471–9. 10.1080/02699050400025059 16134735

[15] Yurkewicz L Weaver J Bullock MR Marshall LF. The effect of the selective NMDA receptor antagonist traxoprodil in the treatment of traumatic brain injury. J Neurotrauma. (2005) 22:1428–43. 10.1089/neu.2005.22.1428 16379581

[16] Cigel A Sayin O Gurgen SG Sonmez A. Long term neuroprotective effects of acute single dose MK-801treatment against traumatic brain injury in immature rats. Neuropeptides. (2021) 88:102161. 10.1016/j.npep.2021.102161 34098454

[17] Ziebell JM Morganti-Kossmann MC. Involvement of pro-and anti-inflammatory cytokines and chemokines in the pathophysiology of traumatic brain injury. Neurotherapeutics. (2010) 7:22–30. 10.1016/j.nurt.2009.10.016 20129494 PMC5084109

[18] Simon DW Mcgeachy MJ Bayir H Clark RS Loane DJ Kochanek PM. The far-reaching scope of neuroinflammation after traumatic brain injury. Nature Rev Neurol. (2017) 13:171–91. 10.1038/nrneurol.2017.13 28186177 PMC5675525

[19] Yan EB Frugier T Lim CK Heng B Sundaram G Tan M. Activation of the kynurenine pathway and increased production of the excitotoxin quinolinic acid following traumatic brain injury in humans. J Neuroinflammation. (2015) 12:1–17. 10.1186/s12974-015-0328-2 26025142 PMC4457980

[20] Brundin L Sellgren C Lim C Grit J Pålsson E Landén M. An enzyme in the kynurenine pathway that governs vulnerability to suicidal behavior by regulating excitotoxicity and neuroinflammation. Transl Psychiatry. (2016) 6:e865–e865. 10.1038/tp.2016.133 27483383 PMC5022080

[21] Dehhaghi M Kazemi Shariat Panahi H Heng B Guillemin GJ. The gut microbiota, kynurenine pathway, and immune system interaction in the development of brain cancer. Front Cell Dev Biol. (2020) 8:562812. 10.3389/fcell.2020.562812 33330446 PMC7710763

[22] Ting KK Brew B Guillemin G. The involvement of astrocytes and kynurenine pathway in Alzheimer's disease. Neurotox Res. (2007) 12:247–62. 10.1007/BF03033908 18201952

[23] Gulaj E Pawlak K Bien B Pawlak D. Kynurenine and its metabolites in Alzheimer's disease patients. Adv Med Sci. (2010) 55:204–11. 10.2478/v10039-010-0023-6 20639188

[24] Chen Y Stankovic R Cullen KM Meininger V Garner B Coggan S. The kynurenine pathway and inflammation in amyotrophic lateral sclerosis. Neurotox Res. (2010) 18:132–42. 10.1007/s12640-009-9129-7 19921535

[25] Rajda C Galla Z Polyák H Maróti Z Babarczy K Pukoli D. Cerebrospinal fluid neurofilament light chain is associated with kynurenine pathway metabolite changes in multiple sclerosis. Int J Mol Sci. (2020) 21:2665. 10.3390/ijms21082665 32290514 PMC7216195

[26] Saraste M Matilainen M Rajda C Galla Z Sucksdorff M Vécsei L. Association between microglial activation and serum kynurenine pathway metabolites in multiple sclerosis patients. Mult Scler Relat Disord. (2022) 59:103667. 10.1016/j.msard.2022.103667 35151985

[27] Guillemin GJ Kerr SJ Smythe GA Smith DG Kapoor V Armati PJ. Kynurenine pathway metabolism in human astrocytes: a paradox for neuronal protection. J Neurochem. (2001) 78:842–53. 10.1046/j.1471-4159.2001.00498.x 11520905

[28] Chen Y Guillemin GJ. Kynurenine pathway metabolites in humans: disease and healthy states International journal of tryptophan research. IJTR S. (2009) 2:2097. 10.4137/IJTR.S2097 22084578 PMC3195227

[29] Dehhaghi M Panahi HKS Kavyani B Heng B Tan V Braidy N. The role of kynurenine pathway and NAD^+^ metabolism in myalgic encephalomyelitis/chronic fatigue syndrome. Aging & Dis. (2022) 13:0824. 10.14336/AD.2021.0824 35656104 PMC9116917

[30] Prendergast GC Malachowski WJ Mondal A Scherle P Muller AJ. Indoleamine 2, 3-dioxygenase and its therapeutic inhibition in cancer. Int Rev Cell Mol Biol. (2018) 336:175–203. 10.1016/bs.ircmb.2017.07.004 29413890 PMC6054468

[31] Dehhaghi M Kazemi Shariat Panahi H Guillemin GJ. Microorganisms, tryptophan metabolism, and kynurenine pathway: a complex interconnected loop influencing human health status. Int J Tryptophan Res. (2019) 12:1178646919852996. 10.1177/1178646919852996 31258331 PMC6585246

[32] Guillemin GJ Brew BJ. Implications of the kynurenine pathway and quinolinic acid in Alzheimer's disease. Redox Report. (2002) 7:199–206. 10.1179/135100002125000550 12396664

[33] Jones SP Franco NF Varney B Sundaram G Brown DA De Bie J. Expression of the kynurenine pathway in human peripheral blood mononuclear cells: implications for inflammatory and neurodegenerative disease. PLoS ONE. (2015) 10:e0131389. 10.1371/journal.pone.0131389 26114426 PMC4482723

[34] Guillemin GJ Wang L Brew BJ. Quinolinic acid selectively induces apoptosis of human astrocytes: potential role in AIDS dementia complex. J Neuroinflammation. (2005) 2:1–6. 10.1186/1742-2094-2-16 16042813 PMC1187916

[35] Ting KK Brew BJ Guillemin GJ. Effect of quinolinic acid on human astrocytes morphology and functions: implications in Alzheimer's disease. J Neuroinflammation. (2009) 6:1–13. 10.1186/1742-2094-6-36 20003262 PMC2797503

[36] Ruddick JP Evans AK Nutt DJ Lightman SL Rook GA Lowry CA. Tryptophan metabolism in the central nervous system: medical implications. Expert Rev Mol Med. (2006) 8:1–27. 10.1017/S1462399406000068 16942634

[37] Schwarcz R Bruno JP Muchowski PJ Wu H-Q. Kynurenines in the mammalian brain: when physiology meets pathology. Nature Rev Neurosci. (2012) 13:465–77. 10.1038/nrn3257 22678511 PMC3681811

[38] Colpo GD Venna VR Mccullough LD Teixeira AL. Systematic review on the involvement of the kynurenine pathway in stroke: pre-clinical and clinical evidence. Front Neurol. (2019) 778. 10.3389/fneur.2019.00778 31379727 PMC6659442

[39] Isabel Cuartero M De La Parra J Garcia-Culebras A Ballesteros I Lizasoain I. The kynurenine pathway in the acute and chronic phases of cerebral ischemia. Curr Pharm Des. (2016) 22:1060–73. 10.2174/1381612822666151214125950 25248805 PMC4972938

[40] Braidy N Grant R Adams S Brew BJ Guillemin GJ. Mechanism for quinolinic acid cytotoxicity in human astrocytes and neurons. Neurotox Res. (2009) 16:77–86. 10.1007/s12640-009-9051-z 19526301

[41] Guillemin GJ. Quinolinic acid, the inescapable neurotoxin. FEBS J. (2012) 279:1356–65. 10.1111/j.1742-4658.2012.08485.x 22248144

[42] Davalos D Grutzendler J Yang G Kim JV Zuo Y Jung S. ATP mediates rapid microglial response to local brain injury in vivo. Nat Neurosci. (2005) 8:752–8. 10.1038/nn1472 15895084

[43] Alam A Thelin EP Tajsic T Khan DZ Khellaf A Patani R. Cellular infiltration in traumatic brain injury. J Neuroinflammation. (2020) 17:1–17. 10.1186/s12974-020-02005-x 33143727 PMC7640704

[44] Balança B Desmurs L Grelier J Perret-Liaudet A Lukaszewicz A-C. DAMPs and RAGE pathophysiology at the acute phase of brain injury: an overview. Int J Mol Sci. (2021) 22:2439. 10.3390/ijms22052439 33670976 PMC7957733

[45] Tavares RG Tasca CI Santos CE Alves LCB Porciúncula LO Emanuelli T. Quinolinic acid stimulates synaptosomal glutamate release and inhibits glutamate uptake into astrocytes. Neurochem Int. (2002) 40:621–7. 10.1016/S0197-0186(01)00133-4 11900857

[46] Lugo-Huitrón R Ugalde Muñiz P Pineda B Pedraza-Chaverrí J Ríos C Pérez-De La Cruz V. Quinolinic acid: an endogenous neurotoxin with multiple targets. Oxid Med Cell Longev. (2013) 2013:24. 10.1155/2013/104024 24089628 PMC3780648

[47] Guillemin GJ Kerr SJ Brew BJ. Involvement of quinolinic acid in AIDS dementia complex. Neurotox Res. (2005) 7:103–23. 10.1007/BF03033781 15639803

[48] Lim CK Fernandez-Gomez FJ Braidy N Estrada C Costa C Costa S. Involvement of the kynurenine pathway in the pathogenesis of Parkinson's disease. Prog Neurobiol. (2017) 155:76–95. 10.1016/j.pneurobio.2015.12.009 27072742

[49] Sinz EH Kochanek PM Heyes MP Wisniewski SR Bell MJ Clark RS. Quinolinic acid is increased in CSF and associated with mortality after traumatic brain injury in humans. J Cereb Blood Flow Metab. (1998) 18:610–5. 10.1097/00004647-199806000-00002 9626184

[50] Frugier T Morganti-Kossmann MC O'reilly D Mclean CA. In situ detection of inflammatory mediators in post mortem human brain tissue after traumatic injury. J Neurotrauma. (2010) 27:497–507. 10.1089/neu.2009.1120 20030565

[51] Zhang Y Wang L Ren W. Blast-related traumatic brain injury is mediated by the kynurenine pathway. Neuroreport. (2022) 33:569–76. 10.1097/WNR.0000000000001817 35894672

[52] Singh R Savitz J Teague TK Polanski DW Mayer AR Bellgowan PS. Mood symptoms correlate with kynurenine pathway metabolites following sports-related concussion. J Neurol Neurosurg Psychiatry. (2016) 87:670–5. 10.1136/jnnp-2015-311369 26269650

[53] Meier TB Drevets WC Wurfel BE Ford BN Morris HM Victor TA. Relationship between neurotoxic kynurenine metabolites and reductions in right medial prefrontal cortical thickness in major depressive disorder. Brain Behav Immun. (2016) 53:39–48. 10.1016/j.bbi.2015.11.003 26546831 PMC4783304

[54] Meier TB Nitta ME Teague TK Nelson LD Mccrea MA Savitz J. Prospective study of the effects of sport-related concussion on serum kynurenine pathway metabolites. Brain Behav Immun. (2020) 87:715–24. 10.1016/j.bbi.2020.03.002 32147388 PMC7316609

[55] Meier TB España L Nitta ME Teague TK Brett BL Nelson LD. Positive association between serum quinolinic acid and functional connectivity following concussion. Brain Behav Immun. (2021) 91:531–40. 10.1016/j.bbi.2020.11.011 33176183 PMC7769223

[56] Carroll L Cassidy JD Peloso P Borg J Von Holst H Holm L. Prognosis for mild traumatic brain injury: results of the WHO collaborating centre task force on mild traumatic brain injury. J Rehabilit Med. (2004) 36:84–105. 10.1080/16501960410023859 15083873

[57] Cole WR Bailie JM. Neurocognitive and psychiatric symptoms following mild traumatic brain injury. In:Laskowitz D Grant G, editors. Translational Research in Traumatic Brain Injury. Boca Raton, FL: CRC Press/Taylor and Francis Group (2015). 26583174

[58] Meier TB Savitz J. The kynurenine pathway in traumatic brain injury: Implications for psychiatric outcomes. Biol Psychiatry. (2022) 91:449–58. 10.1016/j.biopsych.2021.05.021 34266671 PMC8630076

[59] Weis CN Webb EK Deroon-Cassini TA Larson CL. Emotion dysregulation following trauma: shared neurocircuitry of traumatic brain injury and trauma-related psychiatric disorders. Biol Psychiatry. (2022) 91:470–7. 10.1016/j.biopsych.2021.07.023 34561028 PMC8801541

[60] Frank D Gruenbaum BF Shelef I Zvenigorodsky V Severynovska O Fleidervish I. Blood glutamate scavenging as a novel glutamate-based therapeutic approach for post-traumatic brain injury anxiety and social impairment. Transl Psychiatry. (2023) 13:41. 10.1038/s41398-023-02329-1 36739271 PMC9899234

[61] Myint AM Kim YK Verkerk R Scharpé S Steinbusch H Leonard B. Kynurenine pathway in major depression: evidence of impaired neuroprotection. J Affect Disord. (2007) 98:143–151. 10.1016/j.jad.2006.07.013 16952400

[62] Dantzer R O'connor JC Freund GG Johnson RW Kelley KW. From inflammation to sickness and depression: when the immune system subjugates the brain. Nat Rev Neurosci. (2008) 9:46–56. 10.1038/nrn2297 18073775 PMC2919277

[63] Steiner J Walter M Gos T Guillemin GJ Bernstein H-G Sarnyai Z. Severe depression is associated with increased microglial quinolinic acid in subregions of the anterior cingulate gyrus: evidence for an immune-modulated glutamatergic neurotransmission? J Neuroinflammation. (2011) 8:1–9. 10.1186/1742-2094-8-94 21831269 PMC3177898

[64] Olney J Labruyere J Wang G Wozniak D Price M Sesma M. NMDA antagonist neurotoxicity: mechanism and prevention. Science. (1991) 254:1515–8. 10.1126/science.1835799 1835799

[65] Muller N Myint A-MJ Schwarz M. Kynurenine pathway in schizophrenia: pathophysiological and therapeutic aspects. Curr Pharm Des. (2011) 17:130–6. 10.2174/138161211795049552 21361867

[66] Kozak R Campbell BM Strick CA Horner W Hoffmann WE Kiss T. Reduction of brain kynurenic acid improves cognitive function. J Neuroscience. (2014) 34:10592–602. 10.1523/JNEUROSCI.1107-14.2014 25100593 PMC6802596

[67] Albuquerque EX Schwarcz R. Kynurenic acid as an antagonist of α7 nicotinic acetylcholine receptors in the brain: facts and challenges. Biochem Pharmacol. (2013) 85:1027–32. 10.1016/j.bcp.2012.12.014 23270993 PMC3721521

[68] Beggiato S Antonelli T Tomasini MC Tanganelli S Fuxe K Schwarcz R. Kynurenic acid, by targeting α7 nicotinic acetylcholine receptors, modulates extracellular GABA levels in the rat striatum in vivo. Eur J Neurosci. (2013) 37:1470–7. 10.1111/ejn.12160 23442092

[69] Liu H Zhang X Shi P Yuan J Jia Q Pi C. α7 Nicotinic acetylcholine receptor: a key receptor in the cholinergic anti-inflammatory pathway exerting an antidepressant effect. J Neuroinflammation. (2023) 20:84. 10.1186/s12974-023-02768-z 36973813 PMC10041767

[70] Serafini G Adavastro G Canepa G Capobianco L Conigliaro C Pittaluga F. Abnormalities in kynurenine pathway metabolism in treatment-resistant depression and suicidality: a systematic review. CNS & Neurol Dis. (2017) 16:440–53. 10.2174/1871527316666170413110605 28412922

[71] Qin Y Wang N Zhang X Han X Zhai X Lu Y. IDO and TDO as a potential therapeutic target in different types of depression. Metab Brain Dis. (2018) 33:1787–800. 10.1007/s11011-018-0290-7 30014175

[72] Dolšak A Gobec S Sova M. Indoleamine and tryptophan 2, 3-dioxygenases as important future therapeutic targets. Pharmacol Ther. (2021) 221:107746. 10.1016/j.pharmthera.2020.107746 33212094

[73] Berman RM Cappiello A Anand A Oren DA Heninger GR Charney DS. Antidepressant effects of ketamine in depressed patients. Biol Psychiatry. (2000) 47:351–4. 10.1016/S0006-3223(99)00230-9 10686270

[74] Kopra E Mondelli V Pariante C Nikkheslat N. Ketamine's effect on inflammation and kynurenine pathway in depression: a systematic review. J Psychopharmacol. (2021) 35:934–45. 10.1177/02698811211026426 34180293 PMC8358579

[75] Parrott J Redus L Santana-Coelho D Morales J Gao X O'connor J. Neurotoxic kynurenine metabolism is increased in the dorsal hippocampus and drives distinct depressive behaviors during inflammation. Transl Psychiatry. (2016) 6:e918–e918. 10.1038/tp.2016.200 27754481 PMC5315555

[76] Hokari M Wu H-Q Schwarcz R Smith QR. Facilitated brain uptake of 4-chlorokynurenine and conversion to 7-chlorokynurenic acid. Neuroreport. (1996) 8:15–8. 10.1097/00001756-199612200-00004 9051744

